# Distribution and clinical significance of peripheral blood lymphocyte PD-1 and PD-L1 in non-Hodgkin lymphoma: a retrospective study in Chinese patients

**DOI:** 10.3389/fonc.2026.1683486

**Published:** 2026-02-12

**Authors:** Lu Liu, Lei Wang, Wei Wang, Yansu Wang, Yanrong Wang, Na Xu, Na Li, Haifeng Liu

**Affiliations:** 1 Department of Lymphoma and Hematology, Jilin Cancer Hospital, Changchun, China; 2 Department of Nuclear Medicine, Jilin Cancer Hospital, Changchun, China; 3 Department of Organization of Personnel Section, Jilin Cancer Hospital, Changchun, China; 4 Department of Big Data Center of Clinical, Jilin Cancer Hospital, Changchun, China; 5 The Party Committee of the Institute, Jilin Cancer Hospital, Changchun, China

**Keywords:** immune checkpoint, non - Hodgkin lymphoma lymphoma, PD-1, PD-L1, survival

## Abstract

**Background:**

Programmed death-1 (PD-1) and its ligand PD-L1 play critical roles in immune checkpoint regulation. This study investigated the relationship between PD-1 and PD-L1 expression on peripheral blood lymphocytes and Non- Hodgkin Lymphoma progression.

**Methods:**

This retrospective study included patients diagnosed with Non- Hodgkin Lymphoma at Jilin Cancer Hospital between October 2013 and May 2017. PD-1 and PD-L1 expression on CD4+ and CD8+ T cells was analyzed using flow cytometry.

**Results:**

A total of 125 non- Hodgkin Lymphoma patients (65 males and 60 females; median age, 57 years; range, 14–86 years) were included. PD-1 and PD-L1 expression on CD8+ T cells was significantly higher in patients with elevated lactate dehydrogenase (LDH) (>245 U/L) than in those with normal LDH (PD-1: 16.46% *vs*. 12.68%, P = 0.005; PD-L1: 9.96% *vs*. 7.90%, P = 0.043). PD-1 expression on CD8+ T cells was also higher in patients with bone marrow involvement compared with those without (17.62% *vs*. 14.56%, P = 0.039). Furthermore, PD-L1 expression on both CD4+ and CD8+ T cells was significantly lower in patients who achieved complete response (CR) compared with those with non-CR (CD4+: 11.14% *vs*. 18.61%, P<0.001; CD8+: 7.19% *vs*. 10.60%, P = 0.032). Notably, a decrease in CD4+ PD-1 levels after treatment showed a strong trend towards improved overall survival compared with cases showing increased levels (median survival not reached in the decreased group *vs*. 41.77 months in the increased group, P = 0.066).

**Conclusion:**

Peripheral blood lymphocyte PD-1 and PD-L1 expression are associated with LDH levels, bone marrow involvement, and treatment response. A reduction in CD4+ PD-1 levels after treatment is associated with a trend towards improved overall survival.

## Background

Non-Hodgkin lymphoma ranks among the ten most common malignancies in China, with an age-standardized incidence rate of 3.75 cases per 100,000 people (1, 2). It is also one of the leading causes of cancer-related deaths in the country (3). Management of non-Hodgkin lymphoma involves various approaches tailored to the specific type and stage of the disease, including chemotherapy, radiation therapy, immunotherapy, targeted therapy, and stem cell transplantation (4–7). However, due to the limited understanding of non-Hodgkin lymphoma pathogenesis, chemotherapy remains the primary treatment option, apart from the use of rituximab in CD20-positive B-cell lymphoma (6). Many patients either develop resistance to initial chemotherapy or relapse after remission, leaving limited treatment options and resulting in poor prognoses (5–7). Consequently, identifying new therapeutic targets and predictors of treatment efficacy is critical for improving non-Hodgkin lymphoma management and patient outcomes.

The tumor microenvironment and the immune response to cancer cells play significant roles in controlling tumor progression (8). However, cancer cells can exploit various immune checkpoints to evade detection by the immune system (9). As a result, recent treatment paradigms have shifted toward targeting these immune evasion mechanisms (10). Programmed cell death-1 (PD-1) and its ligand PD-L1 are key immune checkpoints targeted by immunotherapy (11). The PD-1 receptor protein inhibits T cell-mediated immune responses (12). Tumor and stromal cells within the microenvironment can express PD-L1 (B7-H1) and PD-L2 (B7-DC), which bind to PD-1 and suppress lymphocyte proliferation and cytokine production (11, 12). Nivolumab, an anti-PD-1 antibody, was approved by the Food and Drug Administration (FDA) in 2016 for relapsed/refractory Hodgkin’s lymphoma (rrHL) following brentuximab vedotin and autologous stem cell transplantation (ASCT) failure (13). Pembrolizumab, another anti-PD-1 antibody, received FDA and European Commission approval for rrHL in 2017 (14). Beyond their role in checkpoint inhibition, PD-1 and PD-L1 expression may also influence chemotherapy response in lymphoma. Chemotherapy can alter the tumor microenvironment and induce PD-L1 expression on tumor cells, thereby reducing T cell activity and weakening the antitumor immune response (15, 16).

While these therapies show great promise, overall response rates to immune checkpoint blockade are 60%–80% for rrHL but only 20%–40% for non-Hodgkin lymphoma (NHL) (17, 18). In comparison, chemotherapy response rates remain around 50% (19, 20). Thus, reliable methods are needed to identify patient subsets most likely to benefit from these therapies. Tumor tissue provides valuable biomarker information, but its use is limited by restricted availability, tumor heterogeneity, dynamic expression patterns, and technical challenges (21, 22). In contrast, liquid biopsies, such as peripheral blood sampling, offer a convenient, non-invasive means of dynamic monitoring with the advantage of reflecting systemic heterogeneity (22). Previous studies suggest that upregulation of PD-1/PD-L1 expression on peripheral blood cells is closely associated with cancer (23). Moreover, CD28 expression by peripheral blood PD-1+ CD8+ T cells has been proposed as a potential biomarker for predicting responses to PD-1/PD-L1 checkpoint blockade therapy (24). However, the precise roles of PD-1 and PD-L1 expression on peripheral blood immune cells in non-Hodgkin lymphoma remain unclear.

This study aims to investigate the impact of PD-1 and PD-L1 expression on peripheral blood non-Hodgkin lymphocytes in relation to lymphoma progression. The findings may help in developing strategies to identify patients most likely to benefit from anti-PD-1 and anti-PD-L1 therapies.

## Materials and methods

### Study population

This retrospective study included patients diagnosed with non-Hodgkin lymphoma at Jilin Cancer Hospital between October 2013 and May 2017 Eligible patients met the following criteria: (1) diagnosis confirmed by the hospital’s pathology department based on the WHO classification of hematopoietic and lymphoid tissue tumors using morphological and immunohistochemical methods; (2) availability of complete clinical data, treatment records, and follow-up information; (3) newly diagnosed cases without prior anti-lymphoma therapy; and (4) pretreatment peripheral blood PD-1 and PD-L1 test results. Patients were excluded if they had incomplete data, a history of other malignancies, congenital immunodeficiency, infectious diseases, or significant diseases of the heart, liver, kidneys, or other vital organs.

The study was approved by the Ethics Committee of Jilin Cancer Hospital (Approval No. 202507-004-01). The committee waived the requirement for individual informed consent due to the retrospective nature of the study. This study initially included patients with both Hodgkin Lymphoma (HL) and Non-Hodgkin Lymphoma (NHL). Given the fundamental differences in the tumor microenvironment, cell of origin, and standard treatment approaches between HL and NHL, and the small number of HL cases (n=149), the subsequent analyses of biomarker association and prognosis were focused on the NHL cohort (n=125) to ensure biological and clinical homogeneity.

PD-1/PD-L1 status was assessed using fasting venous blood samples collected in EDTA tubes and stored at 4 °C. During the study period, flow cytometry was performed with mouse anti-human monoclonal antibodies, including PD-1 (CD279-PE), PD-L1 (CD274-PE), and CTLA-4 (CD152-PerCP), as well as CD3, CD4, CD25, and CD8. The frequencies of PD-1 and PD-L1 expression were evaluated in all participants using a BD flow cytometer, with a minimum diagnostic threshold of 250,000 events per sample.

### PD-1/PD-L1 flow cytometry analysis

PD-1/PD-L1 status was assessed using fasting venous blood samples collected in EDTA tubes and stored at 4°C. During the study period, flow cytometry was performed with mouse anti-human monoclonal antibodies, including PD-1 (CD279-PE), PD-L1 (CD274-PE), and CTLA-4 (CD152-PerCP), as well as CD3, CD4, CD25, and CD8. Lymphocytes were first gated based on forward and side scatter characteristics. Subsequently, CD3+ T cells were selected. From the CD3+ population, CD4+ and CD8+ subsets were identified. PD-1 (CD279) and PD-L1 (CD274) expression were then analyzed within these CD4+ and CD8+ gates. Isotype controls were used to define positive populations. The frequencies of PD-1 and PD-L1 expression were evaluated in all participants using a BD flow cytometer, with a minimum diagnostic threshold of 250,000 events per sample.

### Data collection

Demographic data (sex and age), clinical characteristics (pathological type, extranodal involvement, bone marrow involvement, stage, β2-microglobulin [β2MG], Eastern Cooperative Oncology Group performance status [ECOG PS], B symptoms, and lactate dehydrogenase [LDH]), PD-1/PD-L1 expression status, treatment response, and complications (leukopenia, neutropenia, anemia, thrombocytopenia, and infection) were extracted from medical records.

Treatment regimens were standardized based on lymphoma subtype. Patients with Diffuse Large B-Cell Lymphoma (DLBCL) received R-CHOP (rituximab, cyclophosphamide, doxorubicin, vincristine, prednisone). Patients with Hodgkin Lymphoma received ABVD (doxorubicin, bleomycin, vinblastine, dacarbazine). Patients with peripheral T-cell lymphoma received CHOP-based regimens. Response (CR/PR) was assessed after 4–6 cycles of these first-line therapies.

Staging was performed in accordance with the 2014 Lugano staging system (25) (**Appendix 1**). Treatment response was evaluated based on the 2014 Lugano criteria for lymphoma treatment response assessment, which include complete response (CR), partial response (PR), stable disease (SD), and progressive disease (PD) (25). Hematologic adverse events were graded according to the Common Terminology Criteria for Adverse Events (CTCAE) version 4.03.

Follow-up data were obtained from the medical records. For patients who remained alive, follow-up was censored on October 31, 2024. The cause and time of death were recorded. Overall survival was calculated from the date of cancer diagnosis to death from any cause.

Serum lactate dehydrogenase (LDH) levels were measured as part of routine clinical biochemistry using an automated analyzer (specify model, e.g., Beckman Coulter AU5800) at the hospital’s central laboratory. The upper limit of normal (ULN) was defined as 245 U/L according to the laboratory’s reference range. Detailed flow cytometry protocol: Whole blood was stained within 4 hours of collection. The antibody panel included: anti-CD3-FITC (clone SK7, BioLegend), anti-CD4-PerCP (clone SK3, BD Biosciences), anti-CD8-APC (clone SK1, BioLegend), anti-PD-1-PE (clone EH12.2H7, BioLegend), and anti-PD-L1-PE/Cy7 (clone 29E.2A3, BioLegend). Appropriate isotype controls were used. Red blood cells were lysed using BD FACS Lysing Solution. A minimum of 100,000 lymphocyte-gated events were acquired on a BD FACS Canto II flow cytometer and analyzed using FlowJo software.

### Statistical analysis

All data were analyzed using SPSS version 26.0 (IBM, Armonk, NY, USA). Categorical variables were expressed as n (%), and non-normally distributed continuous variables were presented as medians with interquartile ranges. The Mann-Whitney U test was used for comparisons between groups, and the Wilcoxon signed-rank test was applied for pre- and post-treatment comparisons. Survival curves were generated using the Kaplan-Meier method and analyzed with the log-rank test, while survival rates were calculated with the life table method. Multivariable Cox stepwise regression was performed to adjust for confounding variables and to identify factors independently associated with overall survival as the dependent variable. Variables with P < 0.10 in univariable analysis were entered into a multivariable Cox proportional hazards model using a backward stepwise elimination procedure (removal criterion P > 0.05). Two-sided P-values <0.05 were considered statistically significant. P-values are reported to two or three decimal places.

## Results

### Characteristics of the patients

A total of 125 patients diagnosed with non-Hodgkin lymphoma were included in this study, comprising 65 males and 60 females, with a median age of 57 years (range, 14–86 years). All patients had an Eastern Cooperative Oncology Group performance status (ECOG PS) of 0–2. The clinical characteristics of the patients are presented in **Table 1**.

**Table 1:** Characteristics of 125 patients with non-Hodgkin lymphoma.

Characteristics	n	%
Sex		
Male	65	52.0
Female	60	48.0
Age		
≤60	78	62.4
>60	47	37.6
Pathological type		
B cell non-Hodgkin lymphoma	92	73.6
T cell non-Hodgkin lymphoma	33	26.4
Extranodal involvement		
No	28	22.4
Yes	97	77.6
Bone marrow involvement		
No	110	88.0
Yes	15	12.0
Stage		
I-II	54	43.2
III-IV	71	56.8
β2-microglobulin		
Normal	60	48.0
Elevated	65	52.0
Lactic dehydrogenase		
Normal	75	60.0
Elevated	50	40.0
B symptoms		
A	91	72.8
B	34	27.2

The associations between baseline clinical characteristics and the expression of peripheral blood lymphocyte PD-1 and PD-L1 in non-Hodgkin lymphoma are illustrated in **Table 2**. No significant associations were observed between PD-1 and PD-L1 expression and clinical parameters such as sex, age, disease status, pathological type, β2-microglobulin (β2MG) levels, ECOG PS, B symptoms, extranodal involvement, and bone marrow involvement. However, PD-1 expression on CD8+ T cells was significantly higher in patients with elevated LDH levels (>245 U/L) than in those with normal LDH levels (PD-1: 16.46 [12.40-23.96]% *vs*. 12.68 [10.54-16.84]%, P = 0.005), as shown in **Table 2**.

**Table 2:** Association between PD-1/PD-L1 expression and clinical characteristics (n=125).

Characteristics	n	PD1-CD4(%), median (IQR)	P	PD1-CD8(%), median (IQR)	P	PDL1-CD4(%), median (IQR)	P	PDL1-CD8(%), median (IQR)	P
Sex									
Male	65	22.30 (17.38-26.03)	0.169	14.32 (11.22-17.25)	0.974	12.67 (10.61-22.53)	0.857	8.33 (5.34-13.28)	0.931
Female	60	22.85 (18.81-28.02)		14.87 (10.49-22.39)		14.65 (10.15-21.94)		9.66 (5.69-13.15)	
Age									
≤60	78	22.69 (19.43-27.23)	0.399	14.95 (10.58-21.32)	0.509	13.66 (10.68-21.49)	0.872	8.36 (5.60-13.03)	0.689
>60	47	22.00 (18.56-26.95)		12.84 (10.54-21.94)		14.23 (9.98-22.71)		9.64 (4.69-13.25)	
Pathological type									
B cell NHL	92	22.46 (18.66-26.95)	0.555	13.82 (10.56-21.41)	0.345	14.00 (10.48-22.25)	0.940	9.32 (4.99-12.54)	0.291
T cell NHL	33	22.44 (17.02-27.07)		15.65 (11.62-21.45)		13.54 (10.34-21.76)		9.67 (6.67-15.36)	
β2MG									
Normal	60	21.85 (18.56-25.89)	0.163	14.87 (10.58-23.56)	0.580	13.66 (10.47-22.36)	0.935	9.09 (5.90-12.13)	0.548
Elevated	65	23.25 (19.18-27.05)		13.87 (10.54-19.48)		14.23 (10.35-21.66)		9.50 (5.07-13.61)	
LDH									
Normal	75	22.15 (18.56-26.95)	0.338	12.68 (10.54-16.84)	0.005*	13.44 (9.68-22.15)	0.412	8.33 (4.46-12.28)	0.070
Elevated	50	22.74 (19.09-27.80)		16.46 (12.40-23.96)		15.07 (10.97-22.20)		10.30 (6.32-15.05)	
Stage									
0-II	54	21.99 (18.19-26.95)	0.180	13.28 (10.54-18.12)	0.103	13.11 (10.01-22.20)	0.699	10.16 (4.59-12.72)	0.690
III-IV	71	22.78 (19.68-21.70)		15.51 (10.98-22.54)		14.22 (10.75-21.96)		8.66 (5.87-13.58)	
B symptoms									
A	91	22.44 (19.67-26.95)	0.504	14.32 (10.58-21.61)	0.822	14.23 (10.54-21.54)	0.393	9.68 (5.71-13.63)	0.127
B	34	22.50 (15.69-27.33)		15.26 (10.50.-20.60)		12.91 (8.94-22.20)		6.86 (5.17-11.61)	
Extranodal involvement									
No	28	23.30 (19.48-27.10)	0.292	140.4 (10.55-18.75)	0.700	12.31 (9.95-19.86)	0.492	7.02 (4.83-10.64)	0.175
Yes	97	22.36 (18.60-26.95)		14.88 (10.58-21.70)		14.28 (10.50-22.25)		9.67 (5.93-13.66)	
Bone marrow involvement									
No	110	22.34 (18.56-26.95)	0.185	13.94 (10.55-20.21)	0.053	14.00 (9.93-21.62)	0.179	9.44 (5.31-12.86)	0.521
Yes	15	22.78 (20.84-29.67)		16.84 (12.84-29.67)		13.64 (10.97 + 33.10)		9.67 (6.18-15.25)	

Values are median (IQR) NHL, non-Hodgkin lymphoma; β2MG, β2-microglobulin; LDH, lactate dehydrogenase.

### Patients with CR demonstrated lower PD-L1 expression

All patients underwent chemotherapy, and treatment responses were assessed in 103 individuals (82.4%). Among these evaluable patients, 34(33.0%) achieved CR, 62(60.2%) achieved PR, 6 (5.8%) experienced SD, and 1 patient exhibited PD. Notably, PD-L1 expression on CD4+ and CD8+ T cells was significantly lower in patients with CR compared to those without CR (CD4+: 11.14% [5.98-19.09] *vs*. 18.61% [1.63-23.55], P < 0.001; CD8+: 7.19% [4.21-10.65] *vs*. 10.60% [6.15-15.17], P = 0.032. PD-1 expression did not show any significant association with treatment response (**Table 3**).

**Table 3:** PD-1/PD-L1 expression and treatment response (n=103).

Response	n	PD1-CD4(%), median (IQR)	P	PD1-CD8(%), median (IQR)	P	PDL1-CD4(%), median (IQR)	P	PDL1-CD8(%), median (IQR)	P
CR	34	22.57 (18.56-27.36)	0.978	14.57 (11.10-18.45)	0.883	11.14 (5.98-19.09)	0.001*	7.19 (4.21-10.65)	0.032*
Non-CR	69	22.31 (18.66-26.97)		13.87 (10.56-23.79)		18.61 (11.63-23.55)		10.60 (6.15-15.17)	

IQR, interquartile range; CR, complete response.

### Adverse reactions

Adverse reactions were documented in all patients, including hematological toxicity and infection. PD-1 expression on CD8+ T cells was found to be elevated in patients experiencing more severe leukopenia (15.72 [11.54-23.72]% *vs*. 12.69 [10.52-15.81]%, P = 0.008). Additionally, PD-1 expression on CD4+ T cells was significantly higher in patients with more severe anemia and thrombocytopenia (anemia: 26.25 [22.51-44.17]% *vs*. 22.31 [18.56-26.95]%, P = 0.018; thrombocytopenia:25.40 [20.50-29.56]% *vs*. 22.15 [18.56-26.63]%, P = 0.042) (**Table 4**).

**Table 4:** Association between PD-1/PD-L1 expression and adverse events (n=125).

Adverse reaction grade	n	PD1-CD4(%), median (IQR)	P	PD1-CD8(%), median (IQR)	P	PDL1CD4(%), median (IQR)	P	PDL1CD8(%), median (IQR)	P
Leucopenia									
0-2	47	21.86 (16.99-25.69)	0.140	12.69 (10.52-15.81)	0.008*	12.67 (10.75-21.54)	0.733	9.58 (4.85-13.68)	0.927
3-4	78	22.69 (19.09-27.75)		15.72 (11.54-23.72)		14.25 (10.21-23.03)		8.85 (5.83-12.86)	
Neutropenia									
0-2	39	21.86 (16.99-26.37)	0.364	13.77 (10.52-16.84)	0.204	12.59 (10.44-21.96)	0.685	9.52 (5.32-14.98)	0.649
3-4	86	22.60 (19.09-27.10)		15.50 (11.15-22.79)		14.26 (10.41-22.45)		9.44 (5.60-12.62)	
Anemia									
0-2	114	22.31 (18.56-26.95)	0.018*	14.11 (10.57-20.21)	0.086	13.54 (10.39-22.01)	0.405	8.97 (5.34-12.97)	0.234
3-4	11	26.25 (22.51-44.17)		16.57 (11.67-36.25)		20.28 (11.67-22.36)		10.44 (6.36-29.67)	
Thrombocytopenia									
0-2	101	22.15 (18.56-26.63)	0.042*	14.56 (10.58-20.24)	0.804	13.54 (10.45-21.40)	0.211	9.50 (5.70-12.87)	0.836
3-4	24	25.40 (20.50-29.56)		14.18 (10.71-23.45)		18.00 (7.34-29.22)		8.27 (4.98-14.85)	
Infection									
No	89	22.15 (18.66-26.75)	0.276	13.87 (10.55-20.46)	0.202	12.67 (10.28-21.46)	0.175	8.06 (4.87-12.27)	0.072
Yes	36	23.02 (16.65-29.50)		15.71 (11.77-23.13)		15.82 (10.56-23.56)		10.51 (6.00-15.52)	

IQR, interquartile range.

### Associations of PD-1 and PD-L1 with prognosis

Within a median follow-up period of 94.0 months (range: 0.0–132.0), fore patients (3.2%) were lost to follow-up, and 62 patients (49.6%) died from the disease. **Table 5** presents the results of the univariable and multivariable analyses of prognostic factors for OS. The Cox multivariable analysis revealed that age >60 years (HR: 2.89 95% CI: 1.52-5.52; P=0.001), T cell NHL(HR: 2.90, 95% CI: 1.48-5.69 P = 0.002), stage III–IV disease (HR: 1.95, 95% CI: 1.02-3.71 P=0.043), severe anemia (HR: 2.96 95% CI: 1.28-6.85; P=0.011), and infection (HR: 2.57, 95% CI: 1.37-4.81; P=0.003) were independent predictors of OS. Notably, PD-1 and PD-L1 levels showed no significant association with prognosis.

**Table 5:** Univariable and multivariable analysis of prognostic factors for overall survival (n=125).

Characteristics	Univariable analysis		Multivariable analysis	
	HR (95%CI)	P	HR (95%CI)	P
Sex				
Male	1			
Female	0.90 (0.54-1.51)	0.690		
Age				
≤60	1		1	
>60	2.21 (1.32-3.71)	0.003*	2.89 (1.52-5.52)	0.001*
Pathological type				
B cell NHL	1		1	
T cell NHL	2.56 (1.50-4.40)	0.001*	2.90 (1.48-5.69)	0.002*
Extranodal involvement				
No	1			
Yes	1.29(0.67-2.49)	0.445		
Bone marrow involvement				
No	1			
Yes	2.03 (1.02-4.01)	0.043*		
Stage				
0-II	1		1	
III-IV	2.56 (1.45-4.51)	0.001*	1.95 (1.02-3.71)	0.043*
β2-microglobulin				
Normal	1			
Elevated	2.38 (1.38-4.09)	0.002*		
Lactic dehydrogenase				
Normal	1			
Elevated	1.70 (1.01-2.85)	0.044*		
B symptoms				
A	1			
B	1.06 (0.60-1.87)	0.841		
Response				
CR	1			
Non-CR	2.37 (1.14-4.95)	0.021*		
Leucopenia				
Grade 0-2	1			
Grade 3-4	1.24 (0.72-2.14)	0.432		
Neutropenia				
Grade 0-2	1			
Grade 3-4	1.01 (0.58-1.77)	0.964		
Anemia				
Grade 0-2	1		1	
Grade 3-4	3.09 (1.45-6.57)	0.003*	2.96 (1.28-6.85)	0.011*
Thrombocytopenia				
Grade 0-2	1			
Grade 3-4	1.18 (0.63-2.23)	0.609		
Infection				
No	1		1	
Yes	2.55 (1.51-4.31)	<0.001*	2.57 (1.37-4.81)	0.003*
PD1-CD4 (%)	1.00 (0.97-1.03)	0.926		
PD1-CD8 (%)	1.01 (0.99-1.03)	0.568		
PDL1CD4 (%)	1.02 (0.99-1.04)	0.220		
PDL1CD84 (%)	1.01 (0.98-1.04)	0.470		

HR, hazard ratio; CI, confidence interval; NHL, non-Hodgkin lymphoma; CR, complete response.

a Variables with P < 0.10 in univariable analysis were included in the multivariable Cox model (backward stepwise elimination).

**Figure 1:**
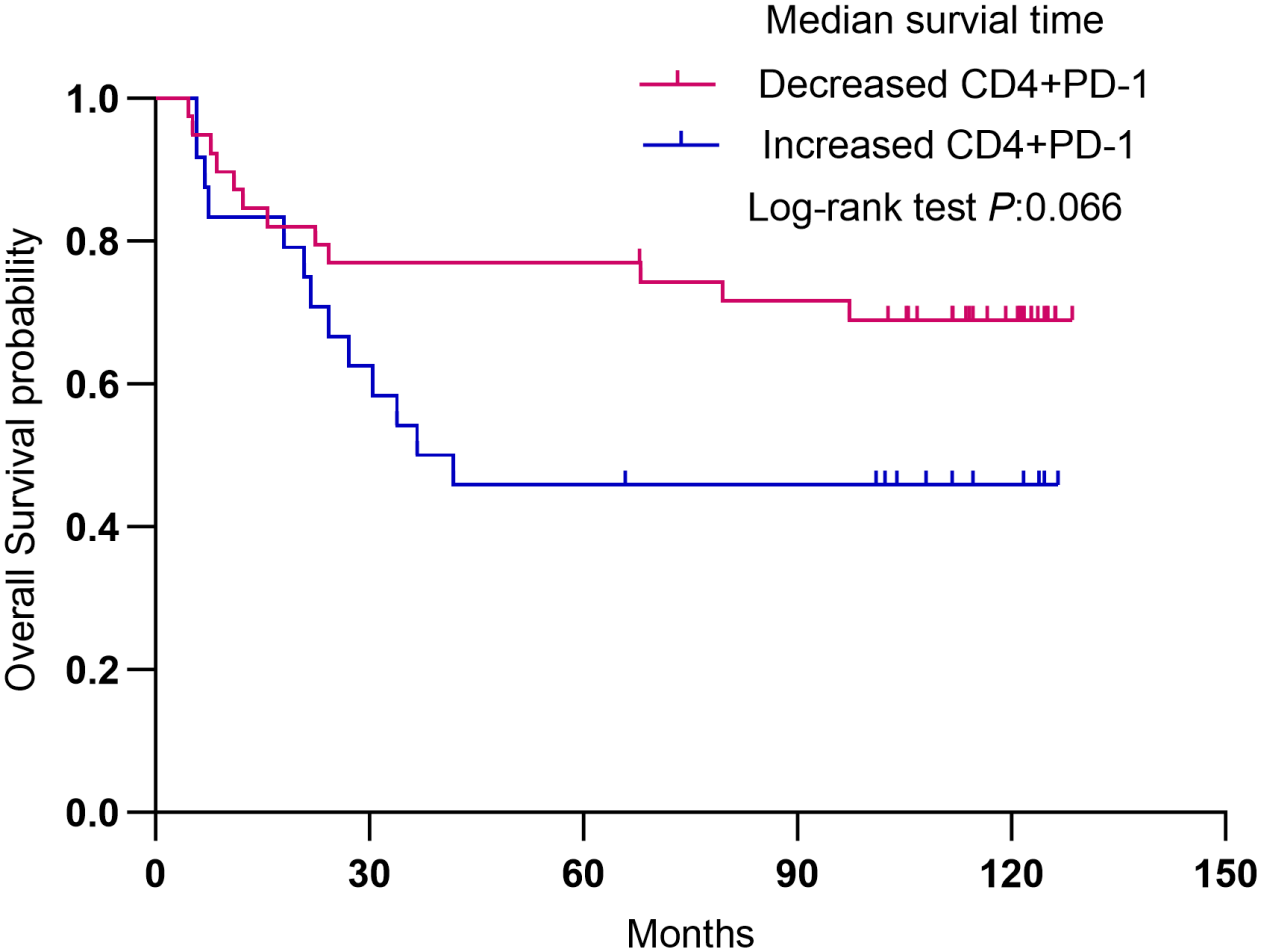
Survival analysis in 63 patients with lymphoma. Overall survival (OS) was analyzed according to the change in PD-1+ CD4 +.

Conversely, changes in PD-1 expression before and after treatment appeared to correlate with outcomes, as patients with decreased PD-1 levels post-treatment had longer OS. Blood samples were collected from 63 patients before and after treatment (57 received anthracycline-based chemotherapy, five received gemcitabine-based chemotherapy, and one received methotrexate-based chemotherapy) (**Table 6**). Two patients (3.2%) were lost to follow-up. Among the remaining patients, 39 (61.9%) demonstrated decreased CD4+ PD-1 levels post-treatment, while 24 patients (38.1%) exhibited an increase. Patients with reduced CD4+ PD-1 levels showed superior survival compared with those with increased levels (rP=0.066) (**Figure 1**).

**Table 6:** Chemotherapy regimens for patients with paired blood samples (n=63).

Characteristics	n	%
Sex		
Male	33	52.4
Female	30	47.6
Age		
≤60	45	71.4
>60	18	28.6
Pathological type		
B cell non-Hodgkin lymphoma	48	76.2
T cell non-Hodgkin lymphoma	15	23.8
Extranodal involvement		
No	19	30.2
Yes	44	69.8
Bone marrow involvement		
No	58	92.1
Yes	5	7.9
Stage		
I-II	32	50.8
III-IV	31	49.2
β2-microglobulin		
Normal	32	50.8
Elevated	31	49.2
Lactic dehydrogenase		
Normal	38	60.3
Elevated	25	39.7
B symptoms		
A	44	69.8
B	19	30.2

## Discussion

This study explored the potential association between PD-1 and PD-L1 expression on peripheral blood lymphocytes and the progression of non-Hodgkin lymphoma. The findings indicate that peripheral blood lymphocyte PD-1/PD-L1 expression is associated with LDH, bone marrow involvement, and treatment response. A reduction in CD4+ PD-1 levels after treatment appeared to be associated with overall survival (OS) benefits. These observations suggest that monitoring peripheral blood lymphocyte PD-1 and PD-L1 could provide insights into treatment response and complications in non-Hodgkin lymphoma patients. Although the results are promising, further prospective studies are required to validate the clinical utility of PD-1/PD-L1 as biomarkers for monitoring disease progression and prognosis in non-Hodgkin lymphoma.

The observed lower peripheral PD-L1 expression on T cells in patients achieving CR likely reflects a reduced state of systemic immune activation and exhaustion following effective tumor debulking. In contrast, patients with PR, SD, or PD have persistent disease, which continuously presents tumor antigens (47). This chronic antigenic stimulation is a primary driver of T-cell exhaustion, characterized by the upregulation of inhibitory receptors like PD-1 and its ligand PD-L1. Persistent PD-L1 expression on CD4+ and CD8+ T cells in the non-CR group may thus serve as a surrogate marker for an ongoing, but ineffective, immune response against residual tumor cells. This exhausted phenotype is associated with impaired T-cell proliferation, cytokine production (e.g., IFN-γ, TNF-α), and cytotoxic function. Therefore, the failure to downregulate this checkpoint axis post-chemotherapy may indicate a tumor microenvironment that remains immunosuppressive and resistant to immune-mediated clearance, explaining the poorer outcomes in this group. Future studies correlating peripheral PD-1/PD-L1 levels with functional assays of T-cell exhaustion and tumor microenvironment analysis are warranted to validate this mechanistic link (48).

A decrease in CD4+ PD-1 after treatment was associated with improved overall survival. The results provide evidence that PD-1 and PD-L1 are involved in non-Hodgkin lymphoma prognosis. A decrease in PD-L1 expression after treatment in lymphoma may be a positive prognostic indicator (26, 27). While high PD-L1 expression is often associated with poor outcomes, its reduction suggests a potential response to treatment and may even improve survival. Similar results were observed in lung cancer (28), B-cell lymphoma (29), and DLBCL (30).

While the downregulation of PD-1/PD-L1 on tumor-infiltrating lymphocytes or soluble PD-L1 post-treatment has been documented in some lymphoma studies (cite relevant studies) (49), our study provides novel evidence that this dynamic change is also detectable and clinically relevant in the peripheral blood compartment. This is significant for two reasons. First, it supports the use of non-invasive liquid biopsy for monitoring immune checkpoint status. Second, our cohort consisted of patients treated predominantly with conventional chemotherapy, not PD-1/PD-L1 inhibitors. This suggests that effective chemotherapy itself can modulate the systemic immune checkpoint landscape, potentially by reducing tumor antigen burden and associated T-cell exhaustion (50). Our finding that this peripheral decrease is associated with better outcomes highlights its potential as a dynamic, systemic biomarker of effective anti-tumor response, irrespective of the specific therapy used.

The present study revealed that PD-1 and PD-L1 were expressed on peripheral blood CD4+ and CD8+ T cells in patients with non-Hodgkin lymphoma. This differed from a study in small cell lung cancer, in which PD-L1 was not detected (31). The present study also showed that patients with higher LDH levels or bone marrow involvement had higher CD8+ T cell PD-1 and PD-L1 expression compared with those with less severe disease. These findings are consistent with previous studies reporting that PD-1 expression levels on T cells may be positively associated with the progression of gastric cancer, chronic lymphocytic leukemia, and cervical cancer (23, 32, 33). MacFarlane et al. (34) similarly reported increased PD-1 expression on peripheral blood effector T cells in renal cell carcinoma, which was significantly reduced soon after surgical resection of the primary tumor. This could be explained by PD-1 expression being induced by high antigen concentration and prolonged antigen stimulation. Therefore, PD-1 expression in peripheral blood may serve as an immunological predictor of tumor progression and outcomes in lymphoma patients.

Although PD-L1 expression by tumor cells has been used as a biomarker for selecting patients for PD-L1/PD-1 checkpoint blockade therapies, patients whose tumor cells lack PD-L1 expression can still respond positively to such therapies (35). This suggests that PD-L1 expressed by non-malignant cells may contribute to antitumor immunity. In the present study, all patients were treated with chemotherapy. PD-L1 expression on CD4+ and CD8+ T cells was lower in those who achieved complete response (CR) compared with those who did not (i.e., PR, SD, or PD), while PD-1 expression showed no significant association with CR. This finding is supported by Wang et al. (36), who found that patients with extranodal NK/T-cell lymphoma had significantly elevated serum soluble plasma PD-L1 levels if they had increased LDH or failed to achieve CR following chemotherapy. Peripheral blood PD-L1 expression has also been associated with tumor progression and prognosis in gastric cancer, renal cell carcinoma, DLBCL, and multiple myeloma (37–41). On the other hand, in the present study, PD-1 and PD-L1 were not associated with overall survival. PD-L1 on effector CD8+ T cells is required for their survival and function (42), but higher CD8+ PD-L1+ expression here was linked to poorer treatment responses. This may reflect unique characteristics of PD-L1 expression and its role in non-Hodgkin lymphoma, requiring further investigation. Nevertheless, PD-L1 expression on T cells may serve as a predictive biomarker and highlight the potential usefulness of alternative immunological therapeutic strategies, including PD-1 axis inhibitors, in lymphoma.

PD-1 expression on CD8+ T cells was higher in patients with more severe leukopenia, while PD-1 expression on CD4+ T cells was higher in those with more severe anemia or thrombocytopenia. PD-1 expression on T cells has been linked to various hematological disorders, including anemia, thrombocytopenia, and leukopenia. Although the underlying mechanisms are not fully understood, PD-1 regulates T cell activity, and its dysregulation may contribute to immune-mediated damage to the hematopoietic system (43–45). PD-1 is strongly expressed on T cells in patients with aplastic anemia, implying a role in immune-mediated destruction of red blood cell precursors (45). PD-1 expression is also increased in CD4+ and CD8+ T cells in patients with active immune thrombocytopenia (ITP), further supporting the involvement of the PD-1/PD-L1 axis in disease pathogenesis (43, 44, 46).

This study has several limitations. First, it was conducted at a single institution and included a relatively small sample size, limiting the generalizability of the findings. Second, the retrospective design constrained the analysis to available patient records, potentially introducing selection or information bias. Additionally, the study assessed only peripheral blood PD-1 and PD-L1 expression without considering tumor-related parameters, limiting associations with tumor characteristics. Finally, all patients received chemotherapy, but the regimens varied, and results may not reflect broader treatment contexts. Therefore, further prospective studies are warranted to validate these findings and to explore the role of PD-1 and PD-L1 in lymphoma across different therapeutic approaches.

## Conclusion

In summary, peripheral blood lymphocyte PD-1 and PD-L1 levels appear to be associated with clinical factors such as LDH levels, bone marrow involvement, treatment-related adverse events, and treatment efficacy in patients with non-Hodgkin lymphoma. Notably, a decrease in CD4+ PD-1 levels following treatment was associated with improved overall survival. Although these findings are promising, further studies are needed to confirm these associations and to explore their potential clinical applications in non-Hodgkin lymphoma management.

## Data Availability

The original contributions presented in the study are included in the article/**Supplementary Material**. Further inquiries can be directed to the corresponding author.
