# Supplementary material for: Distribution and clinical significance of peripheral blood lymphocyte PD-1 and PD-L1 in non-Hodgkin lymphoma: a retrospective study in Chinese patients
Source: Front Oncol. 2026 Feb 12;16:1683486. doi: 10.3389/fonc.2026.1683486 (PMC12935639; doi:10.3389/fonc.2026.1683486)
Supplement: Supplementary file 1 [file Table1.docx]

**Appendix 1**

| **Stage** | **Description** |
| --- | --- |
| **I** | Involvement of a single lymph node region (I) or involvement of a single extranodal organ without lymph node involvement (IE). |
| **II** | Involvement of ≥2 lymph node regions on the same side of the diaphragm (II) may include localized involvement of an extranodal organ in the drainage area of the affected lymph nodes (IIE) (e.g., thyroid involvement with cervical lymph nodes or mediastinal lymph node extension to lung involvement). |
| **II with bulky disease*** | Stage II cases presenting with bulky masses. |
| **Advanced Stage** | |
| **III** | Involvement of lymph node regions on both sides of the diaphragm or involvement of lymph nodes above the diaphragm with spleen involvement (IIIS). |
| **IV** | Involvement of extranodal organs beyond the lymphatic drainage regions (IV). |
